# New Method of Treatment of Elephantiasis

**Published:** 1918-11

**Authors:** 


**New Method of Treatment of Elephantiasis.** M. C. Walther, Le Prog. Med., Mch. 23, has successfully employed in three cases of elephantiasis of the lower limbs, an artificial supplementation of the lymphatic circulation. A small rubber drain, without lateral perforation, is placed by means of a Chassignac trochar in the deep layer of the subcutaneous cellular tissue, short circuiting the zone of arrest of circulation. At the extremities of the tube, it is button-holed through the aponeurosis into the subjacent space and fixed by a linen suture. He mentions a case of elephantiasis of the arm due to a war wound, with cicatrization. The ability to move the arm returned but the oedema persisted. In the cases of elephantiasis of the lower limb, complete cure was apparent at the expiration of 8-20 months.

